# Prevalence of Oral Mucosal Disorders in Diabetes Mellitus Patients Compared with a Control Group

**DOI:** 10.1155/2016/5048967

**Published:** 2016-10-25

**Authors:** José González-Serrano, Julia Serrano, Rosa María López-Pintor, Víctor Manuel Paredes, Elisabeth Casañas, Gonzalo Hernández

**Affiliations:** Department of Oral Medicine and Surgery, School of Dentistry, Complutense University, Madrid, Spain

## Abstract

Chronic hyperglycemia is associated with impaired wound healing and higher susceptibility to infections. It is unclear whether patients with diabetes mellitus (DM) present more oral mucosal disorders compared to control groups. The objectives were to compare (a) the prevalence rates of oral mucosal disorders in the DM and non-DM population and (b) the prevalence rates of specific disorders in the DM and non-DM population. Full-text articles were included if they met the following inclusion criteria: (a) they must be original articles from scientific journals, (b) they must be only cross-sectional studies in English, (c) the prevalence of oral mucosal disorders in DM patients must be evaluated, (d) results must be compared with a healthy control group, and (e) oral mucosal disorders must be specified in DM and non-DM group. All studies showed higher prevalence of oral mucosal disorders in DM patients in relation to non-DM population: 45–88% in type 2 DM patients compared to 38.3–45% in non-DM groups and 44.7% in type 1 DM patients compared to 25% in non-DM population. Tongue alterations and denture stomatitis were the most frequent significant disorders observed. The quality assessment following the Joanna Briggs Institute (JBI) Prevalence Critical Appraisal Tool showed the low quality of the existing studies.

## 1. Introduction

DM is an endocrine disease characterized by a deficit in the production of insulin with consequent alteration of the process of assimilation, metabolism, and balance of blood glucose concentration [1]. It is expected that the number of people with DM worldwide will increase from 171 million in 2000 to 366 million in 2030 [2] or to 642 million in 2040 [1]. Basically, there are two types of DM: type 1 DM (T1DM) and type 2 DM (T2DM) [3].

DM frequently predisposes to oral complications [4]. DM has been associated with higher prevalence and severity of periodontal disease [5], fungal infections [6], alterations in salivary flow rates, and composition or dental caries [7, 8].

An association of diabetes as a risk factor for oral diseases has been discussed in several studies [9, 10]. Some studies found a possible association between DM and potentially malignant disorders such as leukoplakia, erythroplakia, or lichen planus [11–13]. Other studies have observed higher prevalence of tongue alterations [14] or oral manifestations of candidiasis, including rhomboid glossitis, denture stomatitis, or angular cheilitis [15]. Meanwhile, other studies had neither representative samples nor comparison of DM patients with a control group [16].

Considerable debate exists surrounding the issue, if the presence of oral mucosal disorders is greater in DM than in non-DM patients. No systematic review has been performed up to now. Given the lack of systematic knowledge, we have conducted the first systematic review concerning the prevalence of oral mucosal disorders in DM compared to non-DM patients.

The main objectives of this review were (a) to compare the prevalence rates of oral mucosal disorders in DM and non-DM population and (b) to compare the prevalence rates of specific disorders in DM and non-DM population.

## 2. Materials and Methods

We prepared this systematic review by following the Preferred Reporting Items for Systematic Reviews and Meta-Analyses Protocols (PRISMA-P) 2015 statement [17, 18].

### 2.1. Focused Question

Based on the PRISMA guidelines, a focused question was constructed. The addressed focused PICO question (population, intervention, comparison, and outcome) was the following: do diabetes patients have higher prevalence of oral mucosal disorders compared with a control group?

### 2.2. Search Strategy

**Figure 1:**
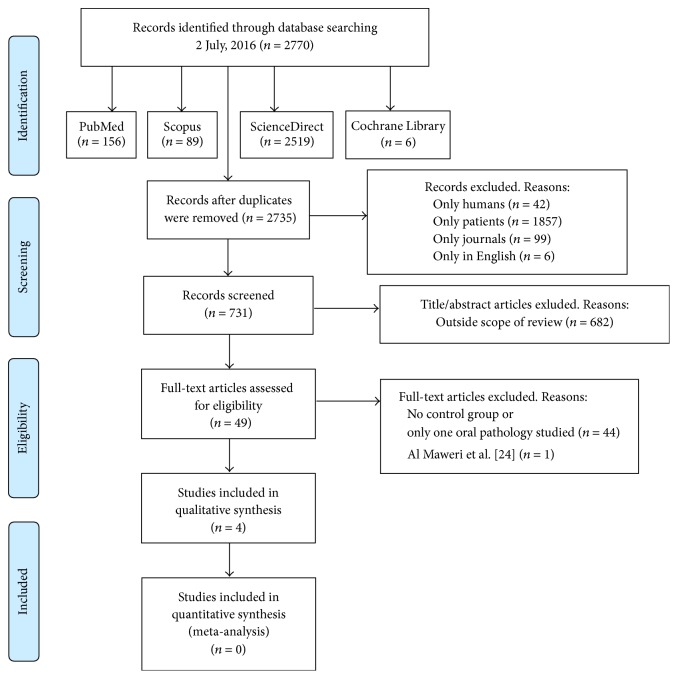
Flow diagram of the literature search, according to the Preferred Reporting Items for Systematic Reviews and Meta-Analyses (PRISMA). PubMed/MEDLINE, Scopus, and ScienceDirect: (diabetes OR “diabetes mellitus”) AND (“oral mucosal lesions” OR “oral diseases” OR “oral pathology”) AND (prevalence OR diagnosis); Cochrane Library: (diabetes OR (diabetes mellitus)) AND ((oral mucosal lesions) OR (oral diseases) OR (oral pathology)) AND (prevalence OR diagnosis).

A comprehensive search of the literature was conducted without date restriction until 2 July 2016 in the following databases: MEDLINE, Scopus, ScienceDirect, and the Cochrane Library. The search strategy used was a combination of Medical Subject Headings (MeSH) terms: (diabetes OR diabetes mellitus) AND (oral mucosal lesions OR oral diseases OR oral pathology) AND (prevalence OR diagnosis) according to each database (Figure 1). Moreover, to ensure completeness of the systematic literature review, an additional hand search to find potential eligible studies was performed and all the references in the articles deemed eligible for inclusion in the study were searched.

### 2.3. Study Selection

#### 2.3.1. Inclusion Criteria

Full-text articles were included regardless of time period of study and year of publication.

*Types of Studies.* The studies had to be (a) original articles published in scientific journals and (b) only cross-sectional studies written in English idiom.

*Types of Population.* Individuals with DM could have T1DM or T2DM. We also considered other diabetes classifications, namely, insulin-dependent DM (IDDM) and non-insulin-dependent DM (NIDDM). A healthy non-DM population as control group must exist.

*Outcomes.* We considered both oral alterations and oral mucosal lesions as disorders. The studies must evaluate the prevalence of oral mucosal lesions or alterations in DM patients. The results must be compared with a healthy control group. The results must specify oral mucosal lesions or alterations in both the DM group and the non-DM group.

#### 2.3.2. Exclusion Criteria

Studies excluded were (a) those published in languages other than English, (b) those studies that compared only one oral pathology (e.g., Lichen planus) to a healthy control group, (c) those studies which were not carried out on humans, and (d) review articles, experimental studies, longitudinal studies, case reports, commentaries, letters to the Editor, and unpublished articles.

### 2.4. Data Collection and Extraction

Two independent researchers (José González-Serrano and Julia Serrano) compared search results to ensure completeness and then duplicates were removed. Those articles not meeting study eligibility criteria using limits such as “only humans,” “only patients,” “only in English,” and “only scientific journals” were also removed. Then the reviewers screened full title and abstracts of the remaining papers individually. Differences in eligible studies were resolved by discussion with a third reviewer (Víctor Manuel Paredes). They went on to obtain the full papers for all potentially eligible studies, which were then checked for eligibility using the standard abstraction forms characteristics, first authors, type of study, country in which study was conducted, recruitment of patients, title of the paper, journal, sample characteristics (population, age, and gender), type of DM, period of time suffering DM, treatment for DM, oral mucosal disorders diagnosis criteria, clinical examination method, clinical observer, and experience (Table 1), and confounding factors such as tobacco, other drugs taken, prosthesis users, DM diagnosis, glycosylated hemoglobin, and diabetic complications (Table 2). The eligible papers were then included in the systematic review. The reported statistical signification was extracted if it was available.

### 2.5. Quality Assessment

The methodological quality in the final selection of eligible studies was evaluated following the Joanna Briggs Institute Prevalence Critical Appraisal Tool [19] (Table 3), which incorporates 10 domains:

1. Was the sample representative of the target population?
2. Were study participants recruited in an appropriate way?
3. Was the sample size adequate?
4. Were the study subjects and the setting described in detail?
5. Was the data analysis conducted with sufficient coverage of the identified sample?
6. Were objective, standard criteria used for the measurement of the condition?
7. Was the condition measured reliably?
8. Was there appropriate statistical analysis?
9. Are all the important confounding factors/subgroups/differences identified and accounted for?
10. Were subpopulations identified using objective criteria?

A study was considered to have a low quality assessment if 0–5 criteria were met and high quality assessment if studies met 5–10 criteria. Two reviewers (Gonzalo Hernández and Rosa María López-Pintor) conducted a critical appraisal independently of each other. The reviewers met to discuss the results of their critical appraisal; if the two reviewers disagreed on the final critical appraisal, a third reviewer (Elisabeth Casañas) was required.

### 2.6. Statistical Methods

The prevalence of oral mucosal disorders from the included studies was presented as a percentage. The results of each oral mucosal disorder were shown along with the number of DM patients and controls, their respective percentages, and their statistical significance when available (Table 4). A meta-analysis was not possible due to the differences between the selected papers: different types of DM, different types of oral disorders, and heterogeneous demographic characteristics (age and ethnic origin).

## 3. Results

### 3.1. Study Selection

The response to the search strategy yielded 2770 results, of which 2735 remained after removing those that were duplicated. We restricted the search to those articles published in English, in humans and patients, and excluded all results that were not published in journals, leaving a total of 731 references. Then, 2 independent researchers (José González-Serrano and Julia Serrano) reviewed all the titles and abstracts, obtaining 49 potential references. Finally, 45 were discarded due to the absence of a control group or because only one selected oral pathology was studied. Only 4 papers were included in our systematic review [20–23] (Figure 1).

Due to similarity between study populations in the papers realized by the groups of Saini et al. [21] and Al Maweri et al. [24], authors were asked if patients of one study were included in another one. The answer was affirmative, proposing us to select only the paper written by the group of Saini et al. [21], since it was more complete.

### 3.2. Study Characteristics

The selected articles were published between 2000 and 2014. A total of 2570 patients were studied, of which 1366 were cases (434 T1DM and 932 T2DM cases) and 1204 were controls. The mean age of the subjects ranged from 33 to 53 years in DM group and from 31 to 51 years in controls. Regarding gender, we studied 1315 women and 1255 men, 673 women and 657 men for DM cases and 606 women and 598 men for the controls (Table 1).

### 3.3. Main Findings

The prevalence of having one or more oral mucosal disorders in T2DM patients was significantly greater than that in the control group according to Saini et al. (45% × 38.3%) [21], Bastos et al. (88% × 45%) [22], and Mohsin et al. (60.8% × 39.2%) [23]. In T1DM patients, the prevalence of having one or more oral disorders was significantly higher than that in the control group (44.7% × 25%) according to Guggenheimer et al. [20].

The types of oral disorders that were found to be statistically significant in more than one of the studies included in DM patients compared with the control group were coated tongue [22, 23], fissured tongue [20, 22, 23], migratory glossitis [21, 22], and denture stomatitis [20, 21]. Every oral disorder found in DM patients and control groups of the selected papers is recorded in Table 4.

### 3.4. Risk of Bias in Individual Studies

Using the predetermined 10 domains for the methodological quality assessment according to the Joanna Briggs Institute Prevalence Critical Appraisal Tool [17], we determined all the selected papers [20–23] to have a low quality assessment (0–5 domains) and none of them to have a high quality assessment (5–10 domains). Table 3 shows a more detailed description of the articles included.

## 4. Discussion

We identified 4 studies reporting prevalence of oral mucosal disorders in DM population compared to non-DM population. Comparisons between studies were limited due to different types of DM, different types of oral disorders, and heterogeneous demographic characteristics (age and ethnic origin) of the studied population. In addition, the quality assessment of studies was low. Hence, no meta-analysis was performed. Nevertheless, there are some patterns that can be described.

In the present systematic review, higher prevalence of oral mucosal disorders was found in patients with DM compared to non-DM patients. This prevalence ranged from 45–88% in T2DM patients to 38.3–45% in non-DM groups and from 44.7% in T1DM patients to 25% in non-DM population. This increased prevalence of oral disorders in DM groups may be due to an inadequate metabolic control of DM or a slow healing process [25]. According to some authors, its cause might be oxidative stress, a decreased antioxidant capacity, or higher levels of inflammatory cytokines, as they are considered as major alternative pathways contributing to the pathogenesis of diabetic complications [26, 27].

Changes of the tongue are more frequent in DM patients than in controls, such as fissured tongue [20, 22, 23], migratory glossitis [21, 22], or coated tongue [22, 23]. There is a strong association between migratory glossitis and fissured tongue [28]. The pathogenesis of fissured tongue is considered to be a genetically determined variant of development, the result of aging, or changes in the oral environment. Migratory glossitis is thought to have hereditary and environmental components [28]. Coated tongue can be associated with a decreased salivary flow present in DM population [9]. These tongue alterations uncommonly require treatment.

DM patients are more susceptible to suffering from fungal infections by* Candida albicans*, especially if they wear prostheses [29]. Guggenheimer et al. [20] and Saini et al. [21] showed that DM patients suffered significantly more denture stomatitis compared to the control groups. Guggenheimer et al. found that the use of dentures was a factor significantly associated with the presence of* Candida* pseudohyphae in T1DM subjects [15]. Thus, diabetes patients using prostheses should have dental check-ups more frequently to prevent this infection. Dental professionals should also provide hygiene measures in order to prevent fungal infections.

Regarding potentially malignant disorders, Bastos et al. found significantly higher prevalence of actinic cheilitis and oral lichen planus in DM patients with regard to the control group [22], while Saini et al. and Mohsin et al. did not find higher prevalence [21, 23]. These findings do not clarify whether there is a need for regular clinical examinations to ensure early diagnosis and treatment of potentially malignant disorders of the oral mucosa in DM patients.

Ujpál et al. saw that smoking diabetes patients are more susceptible to developing leukoplakia [30]. However, tobacco as a confounding factor has not been identified in all studies (Table 2). Guggenheimer et al. only specified tobacco consumption in T1DM patients group [20], Saini et al. excluded tobacco in both groups [21], and Mohsin et al. did not specify this variable [23]. The only authors that included tobacco in both T2DM patients and the control group were Bastos et al., obtaining statistically significant differences in the appearance of nicotine stomatitis in T2DM; nevertheless these authors did not find statistically significant differences of leukoplakia between two groups [22]. Future studies about this topic should take into account this risk factor to establish a possible correlation with the presence of different oral disorders.

A biopsy was performed in three of the four studies included in order to diagnose oral mucosal disorders when required [21–23], but none of them specified how the process was done (fresh tissue for direct immunofluorescence technique or in formaldehyde for a traditional anatomical pathology analysis). It is worth mentioning that none of the selected studies include patients diagnosed with vesiculobullous lesions such as pemphigus vulgaris or benign mucous membrane pemphigoid. However, we do have experience of patients with T2DM and pemphigus vulgaris [31]. Moreover, Heelan et al. in a study of 295 patients diagnosed with different types of pemphigus found that 18% of them were diabetic [32]. The absence of vesiculobullous lesions in the included studies may be due to the absence of direct immunofluorescence diagnostic tests.

Oral hypoglycemics can generate oral and/or skin lichenoid reactions, as seen with tolazamide, tolbutamide, chlorpropamide, glimepiride, or glyburide [33, 34]. It seems strange that none of the studies collected this type of lesions, as they might have classified them as lichen planus. These lesions appear temporarily while taking the drug. Other main drugs taken were collected in three of the four studies [20–22]. In the study of Guggenheimer et al., 2.7% (*p* < 0.05) of T1DM patients were taking immunosuppressive drugs. However, they did not specify how their consumption may influence the occurrence of oral lesions. López-Pintor et al. saw in renal transplant patients under immunosuppressive therapy that the appearance of oral lesions was of 54.7% compared to 19.4% in a healthy control group [35]. For these reasons, it is important to register all drugs taken by patients in order to study a possible connection with oral disorders.

Due to the fact that only articles published in the English language were reviewed, bias due to the language publication could not be ruled out. Although we searched four databases, we cannot guarantee that some related papers might not have been identified. However, we checked the reference lists of reviewed articles to identify relevant studies. The studies reviewed, as we observed previously, presented different types of DM (T1DM and T2DM) which could cause detection bias.

Firstly, none of the included studies specified the blood glucose values that have been used for the diagnosis of DM [20–23]. Only studies by Saini et al. and Mohsin et al. evaluated blood glucose in the control group [21, 23]. Therefore, DM patients could have been present in the control groups of the rest of studies [20, 22]. Secondly, most of the studies did not take into account whether cases of DM are consecutive or not and the observation period. With respect to oral disorders, the type of biopsy taken was unspecified and differing criteria for diagnosing oral mucosal disorders were used, which could also cause bias. Guggenheimer et al. based their diagnosis on onset, duration, oral habits, clinical appearance, history of trauma, and previous episodes [20], Saini et al. based their diagnosis on WHO guide to epidemiology and diagnosis of oral mucosal diseases [21], and the two others did not specify what they based their diagnosis on [22, 23]. Finally, most of studies did not correctly match smoking habit, the use of drugs, and the presence of dentures with oral disorders. These risk factors are very important in some oral disorders etiology.

Prevalence of DM increases with age and T2DM is much more common than T1DM (the latter only accounts for about 10% of DM patients) [36]. Therefore, T2DM population presents greater probability to have oral mucosal disorders. Fungal infections, especially in adult dentures users, will be also easier to find in a daily clinical practice. Thus, periodical oral check-ups should be made in DM population.

## 5. Conclusion

The review conducted demonstrated that the prevalence of oral mucosal disorders in DM patients is statistically higher than that in non-DM individuals. Fungal infections related to dentures (denture stomatitis) and tongue alterations such as coated tongue and fissured tongue or migratory glossitis were the most frequent disorders in the oral cavity. Owing to the high degree of heterogeneity regarding the types of DM, diagnosis of DM, and differing diagnosis criteria of oral disorders, it was difficult to compare the studies. In addition, the quality assessment showed the low quality of the existing studies. Therefore, the results of this systematic review were inconsistent.

We recommend that new studies analyzing the prevalence of oral mucosal disorders in DM population should use more precise and current definitions concerning the determination and diagnosis of DM patients and oral mucosal disorders. New studies should also specify the relationship between the presence of oral disorders and risk factors such as smoking, dentures, and drugs taken by DM patients.

## Supplementary Material

The complete description of how the search was realized for each database is described hereafter. Duplicates were removed introducing all the references found in each database in Refworks and applying “delete duplicates”.

## Figures and Tables

**Table 1:** General characteristics of selected studies.

	Guggenheimer et al., 2000 [20]		Saini et al., 2010 [21]		Bastos et al., 2011 [22]		Mohsin et al., 2014 [23]	
Type of study	Cross-sectional		Cross-sectional		Cross-sectional		Cross-sectional	

Country	USA		Malaysia		Brazil		Pakistan	

Patients recruited at	Department of Oral Medicine, University of Pittsburgh		Endocrinology Clinic of Medical Hospital and Department of Dental School		Clinic of Periodontics, Estadual Paulista University		Baqai Institute of Diabetology and Endocrinology	

Sample	673		840		257		800	
	Cases 405	Controls 268	Cases 420	Controls 420	Cases 146	Controls 111	Cases 395	Controls 405

Age (years)	32.5 ± 0.3		Cases 52.96 ± 10.52	Controls 51.80 ± 11.58	Cases 53.10 ± 7.9	Controls 51.4 ± 10.3	Male 51 ± 8.85 Female 49 ± 8.9	
	Cases 33 ± 0.4	Controls 31.8 ± 0.49					Cases Male 53 ± 9.8 Female 53 ± 8.8	Controls Male 48 ± 7.2 Female 44 ± 5.8

Gender	Male, 312 Female, 361		Male, 352 Female, 488		Male, 109 Female, 148		Male, 482 Female, 318	
	Cases Male, 204 Female, 201	Controls Male, 108 Female, 160	Cases Male, 185 Female, 235	Controls Male, 167 Female, 253	Cases Male, 56 Female, 90	Controls Male, 53 Female, 58	Cases Male, 212 Female, 183	Controls Male, 270 Female, 135

Type of DM	T1DM		T1DM, 29 T2DM, 391		T2DM		T2DM	

Period of time with DM	U		8,36 ± 6.08 years: <5 years: 170 (40,5%) 6–10 years: 138 (32,9%) >10 years: 112 (26,7%)		<10 years: 36 (24.7%) ≥10 years: 110 (75.3%)		U	

Treatment for DM	Insulin 405		Oral hypoglycemics, 274 Insulin, 49 Both, 97		Oral hypoglycemics, 98 (67.1%) Insulin, 29 (19.8%) Both, 19 (13.1%)		U	

Oral mucosal disorders diagnosis criteria	Based on onset, duration, oral habits, clinical appearance, history of trauma, and previous episodes		Based on WHO guide to epidemiology and diagnosis of oral mucosal diseases		U		U	

Biopsy when needed	U		Yes		Yes		Yes	

Clinical examination method	Examination light Dental mirror Gauze square		Electrical overhead light Mouth mirror Tweezers Gauze Wooden tongue depressor		Artificial light Dental mirror Gauze square		Visible light Dental mirror Cotton gauze	

Clinician and experience	2 oral medicine specialists with 10 years of experience		Single examiner assessed by an oral medicine specialist with more than 7 years of experience		Stomatologist		U	

U: unspecified.

**Table 2:** Confounding factors of selected studies.

	Guggenheimer et al., 2000 [20]		Saini et al., 2010 [21]		Bastos et al., 2011 [22]		Mohsin et al., 2014 [23]	
Tobacco	Cases Now, 19.4% Ever, 37.5%	Controls U	Excluded		Cases 25 (17.2%)	Controls 30 (27%)	U	

Other drugs taken	Cases Cardiovascular agents, 19.8%, *p* < 0.01 Immunosuppressants, 2.7%, *p* < 0.05 Anticonvulsants, 2.7%, *p* < 0.05 Thyroid supplements, 8.4%, *p* < 0.001 Antimicrobials, 10.4% Unknown, 5.2%	Controls Cardiovascular agents, 6% Immunosuppressants, 0.4% Anticonvulsants, 0.4% Thyroid supplements, 1.1% Antimicrobials, 8.6% Unknown, 7.1%	Cases Cardiovascular agents, 22.4% Antibiotics, 2.4% NSAID, 3.3% Antiasthmatic drugs, 1.4% Others, 2.4%	Controls Cardiovascular agents, 10% Antibiotics, 1% NSAID, 1.4% Antiasthmatic drugs, 1.7% Others, 1.7%	39.2% taking a daily medication, of which 73.3% were antihypertensives and 56% were antidepressants		U	

Dentures users	Cases Complete or partial dentures, 12.3%, *p* < 0.01	Controls Complete or partial dentures, 3%	U		U		U	

DM diagnosis	U		U	Controls: excluded by fasting blood glucose level	U		U	Controls: excluded by fasting blood glucose level

Glycosylated hemoglobin (HbA1c)	11 ± 0.1		8,49 ± 2,25 Good (<7.5), 172 (41%) Moderate (7.6–8.9), 92 (21.9%) Poor (>9), 156 (37.1%)		Adequate (<7): 38 (26%) Inadequate (≥7): 108 (74%)		U	

Diabetic complications	Nephropathy, 23.2% Neuropathy, 26.9% Retinopathy, 44.4% Peripheral vascular disease, 10.6%		14.5% U		65 (44.5%) Nephropathy, 20.3% Neuropathy, 16.5% Retinopathy, 63.2%		Excluded	

U: unspecified.

**Table 3:** JBI Critical Appraisal Checklist for studies reporting prevalence data.

	Guggenheimer et al., 2000 [20]	Saini et al., 2010 [21]	Bastos et al., 2011 [22]	Mohsin et al., 2014 [23]
(1) Was the sample representative of the target population?	Y	Y	Y	U
(2) Were study participants recruited in an appropriate way?	U	U	U	U
(3) Was the sample size adequate?	U	Y	U	Y
(4) Were the study subjects and setting described in detail?	U	U	U	U
(5) Is the data analysis conducted with sufficient coverage of the identified sample?	U	U	U	U
(6) Were objective, standard criteria used for measurement of the condition?	U	U	N	N
(7) Was the condition measured reliably?	U	U	U	U
(8) Was there appropriate statistical analysis?	Y	Y	Y	Y
(9) Are all the important confounding factors/subgroups/differences identified and accounted for?	N	N	N	N
(10) Were subpopulation identified using objective criteria?	U	Y	Y	U
Total number of “Y”	2	4	3	2
Quality assessment	low	low	low	low

Y: yes; N: no; U: unclear; N/A: not applicable.

**Table 4:** Distribution of oral mucosal disorders in DM patients and controls.

	Guggenheimer et al., 2000 [20]		Saini et al., 2010 [21]		Bastos et al., 2011 [22]		Mohsin et al., 2014 [23]	
	Cases	Controls	Cases	Controls	Cases	Controls	Cases	Controls
	*n* (%)	*n* (%)	*n* (%)	*n* (%)	*n* (%)	*n* (%)	*n* (%)	*n* (%)
Subjects with one or more oral disorders	180 (44.4) *p* < 0.0001	67 (25)	189 (45) *p* < 0.05	161 (38.3)	129 (88) *p* < 0.001	50 (45)	225 (60.8) *p* < 0.0001	145 (39.2)
Angular cheilitis	13 (3.2)	3 (1.1)	10 (2.4) *p* < 0.05	3 (0.7)	22 (15)	10 (9)		
Aphthous stomatitis	6 (1.5)	8 (3.0)	5 (1.2)	3 (0.7)				
Atrophy of tongue papillae	36 (8.9) *p* < 0.001	6 (2.2)			4 (2.7)	0 (0)		
Pseudomembranous candidiasis	2 (0.5)	1 (0.4)						
Denture stomatitis	19 (4.7) *p* < 0.05	4 (1.5)	45 (10.7) *p* < 0.05	26 (6.2)				
Epulis fissuratum	3 (0.7)	0 (0.0)						
Fissured tongue	22 (5.4) *p* < 0.0001	1 (0.4)	114 (27.1)	112 (26.7)	26 (17,8) *p* < 0.001	4 (3.6)	63 (15.9) *p* < 0.05	40 (9.9)
Fistulous tract	4 (1.0)	1 (0.4)						
Gingival hyperplasia	7 (1.7)	4 (1.15)						
Herpes labialis	1 (0.2)	2 (0.7)						
Inflammatory papillary hyperplasia	3 (0.7)	0 (0.0)						
Fibroma	10 (2.5) *p* < 0.05	1 (0.4)	5 (1.2)	5 (1.2)				
Lichen planus	2 (0.5)	2 (0.7)	2 (0.5)	0 (0)	9 (6.1) *p* < 0.01	0 (0)	7 (1.8)	4 (1)
Median rhomboid glossitis	29 (7.2) *p* < 0.0001	1 (0.4)	4 (1)	5 (1.2)				
Geographic tongue	22 (5.4)	9 (3.4)	17 (4) *p* < 0.05	4 (1)	8 (5,4) *p* < 0.01	1 (0,9)	5 (1.3)	4 (1)
Papilloma	1 (0.2)	1 (0.4)						
Traumatic ulcer	14 (3.5) *p* < 0.05	3 (1.1)	8 (1.9)	2 (0.5)				
Frictional keratosis			10 (2.4)	14 (3.3)				
Coated tongue					42 (28,7) *p* < 0.0001	9 (8.1)	106 (26.8) *p* < 0.0001	32 (7.9)
Varices					30 (20,5) *p* < 0.001	6 (5.4)		
Melanin pigmentation					12 (8,2) *p* < 0.01	2 (1,8)	60 (15.2)	45 (11.1)
Leukoedema					8 (5.4)	2 (1.8)		
Actinic cheilitis					37 (25.3) *p* < 0.0001	6 (5.4)		
Leukoplakia					6 (2.7)	1 (1.8)	14 (3.5)	12 (3)
Nicotinic stomatitis					3 (2) *p* < 0.01	2 (1.8)		
Oral submucous fibrosis							8 (2)	12 (3)
Linea alba							31 (7.1) *p* < 0.05	12 (3)
Fordyce granules							9 (2.3)	0 (0)
