# Supplementary material for: Prevalence of Oral Mucosal Disorders in Diabetes Mellitus Patients Compared with a Control Group
Source: J Diabetes Res. 2016 Oct 25;2016:5048967. doi: 10.1155/2016/5048967 (PMC5099460; doi:10.1155/2016/5048967)
Supplement: Supplementary file 1 — The complete description of how the search was realized for each database is described hereafter. Duplicates were removed introducing all the references found in each database in Refworks and applying “delete duplicates”. [file 5048967.f1.docx]

The complete description of how the search was realized for each database is described hereafter. Duplicates were removed introducing all the references found in each database in Refworks and applying “delete duplicates”.


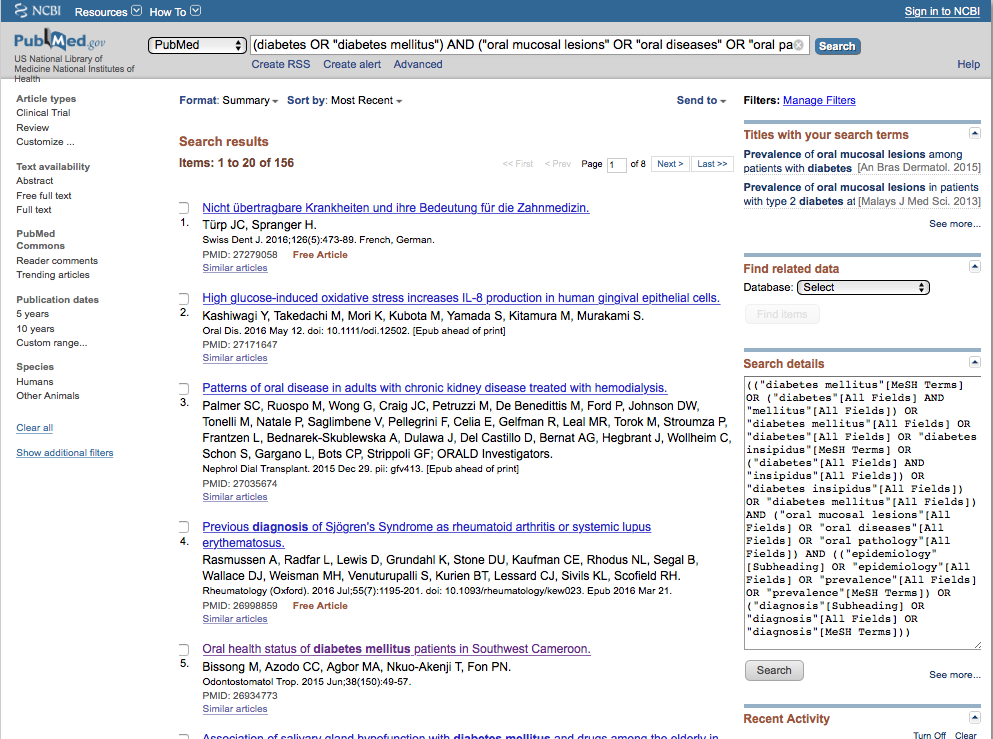


Figure 1. Pubmed/MEDLINE results applying the following search: (diabetes OR "diabetes mellitus") AND ("oral mucosal lesions" OR "oral diseases" OR "oral pathology") AND (prevalence OR diagnosis).


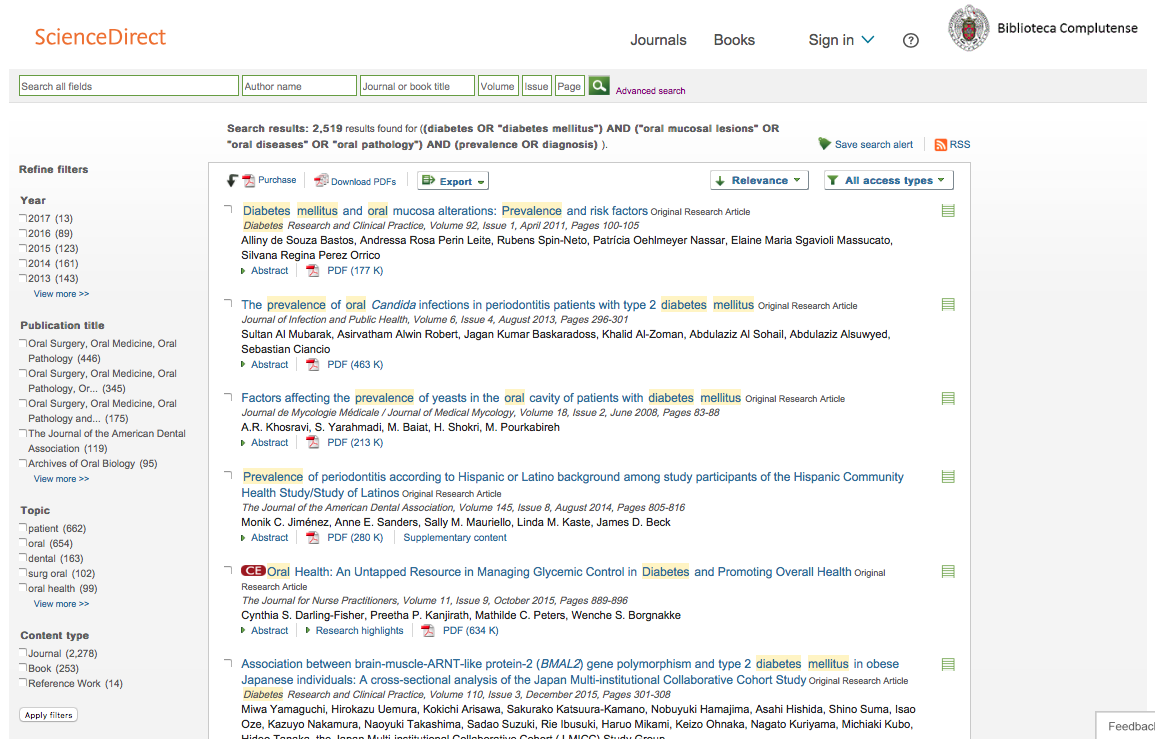


Figure 2. Science Direct results applying the following search: (diabetes OR "diabetes mellitus") AND ("oral mucosal lesions" OR "oral diseases" OR "oral pathology") AND (prevalence OR diagnosis).


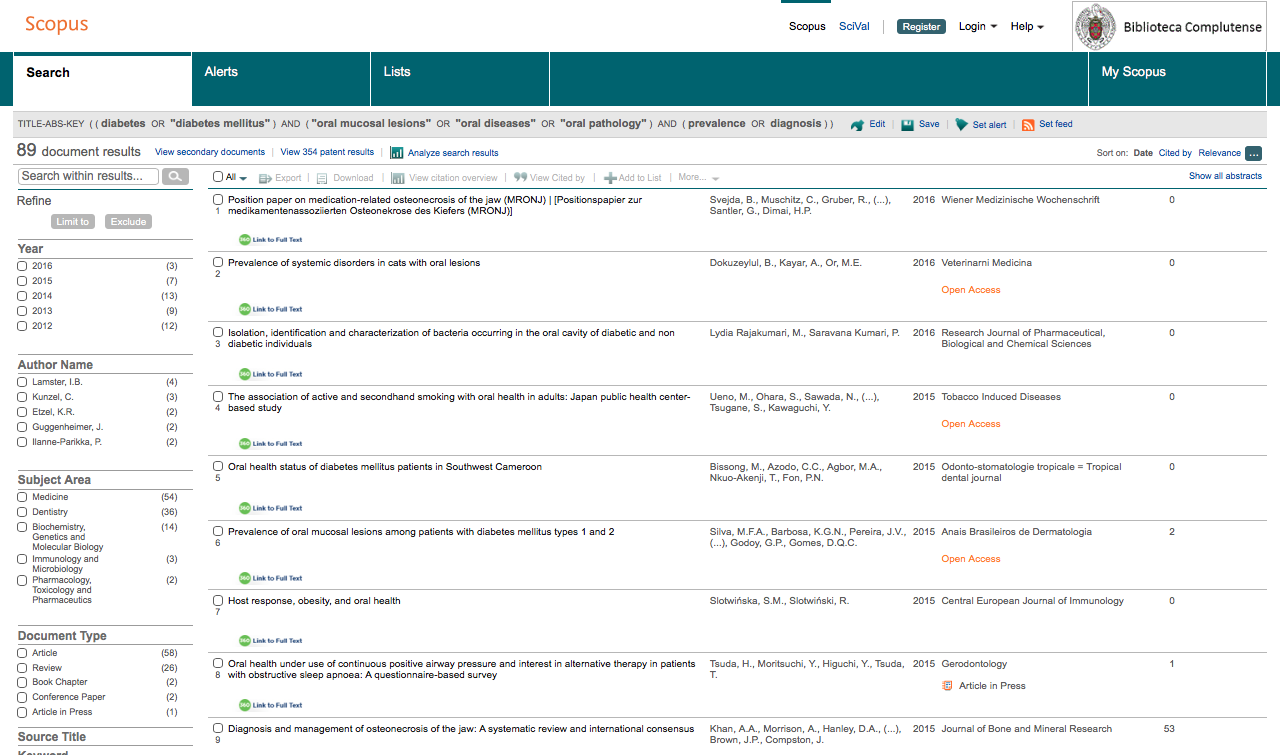


Figure 3. Scopus results applying the following search: (diabetes OR "diabetes mellitus") AND ("oral mucosal lesions" OR "oral diseases" OR "oral pathology") AND (prevalence OR diagnosis).


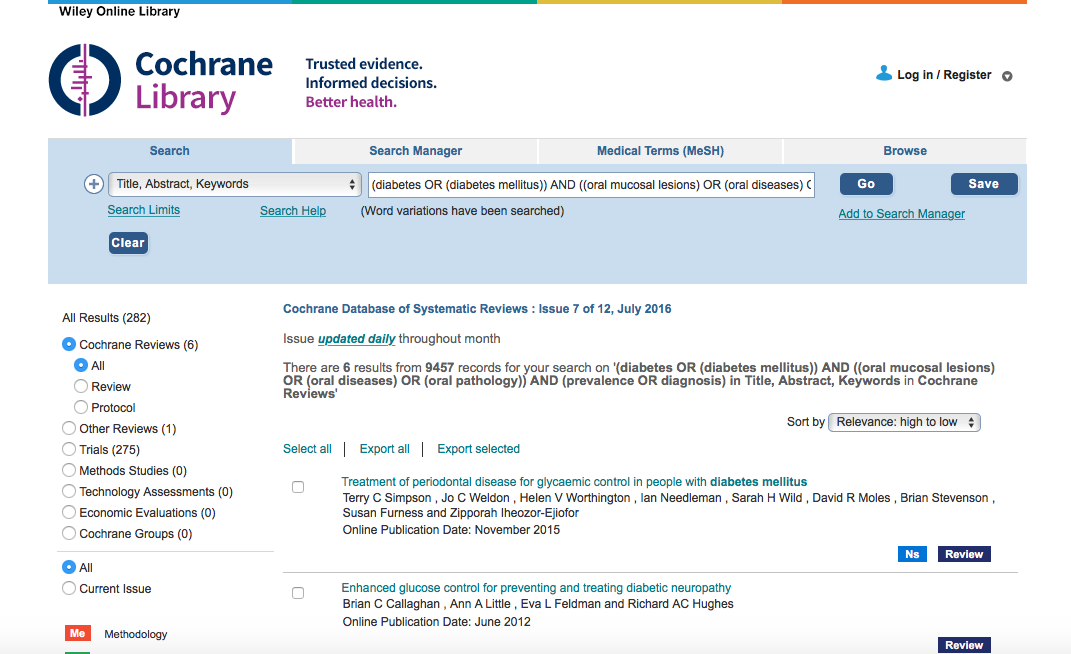


Figure 4. Cochrane Library results applying the following search: (diabetes OR (diabetes mellitus)) AND ((oral mucosal lesions) OR (oral diseases) OR (oral pathology)) AND (prevalence OR diagnosis).
